# Five Years Follow-up of a Spontaneous Eruption of an Impacted Mandibular Premolar Associated with a Dentigerous Cyst Treated by Marsupialization

**DOI:** 10.7759/cureus.7370

**Published:** 2020-03-22

**Authors:** Samia Aboujaoude, Maryse Ziade, Georges Aoun

**Affiliations:** 1 Pediatric Dentistry and Public Dental Health, Lebanese University, Beirut, LBN; 2 Oral Surgery, Lebanese University, Beirut, LBN; 3 Oral Medicine and Maxillofacial Radiology, Lebanese University, Beirut, LBN

**Keywords:** dentigerous cyst, marsupialization, follow-up

## Abstract

Dentigerous cysts (DC) are developmental odontogenic cysts associated with impacted or partially erupted teeth; they can occur at any location of the jaw. Being generally asymptomatic, they are fortuitously discovered when radiographs are taken to investigate a tooth eruption failure. In this report, we present a case of a 10-year-old girl presented with the absence of the right second mandibular premolar and retention of the right second primary molar. After clinical and radiological examinations a preliminary diagnosis of the DC was made and confirmed later histopathologically. The lesion was treated by marsupialization to allow eruption of the affected tooth and followed up for five years with no evidence of recurrence.

## Introduction

A dentigerous cyst (DC) is a developmental odontogenic cyst associated with impacted or partially erupted teeth [1]. It is considered the second most common cyst of the oral cavity after the radicular cyst [2-3].

A DC can occur at any location of the jaw but it is commonly seen in relation to mandibular third molars followed by the maxillary canines and the maxillary third molars [3-6]. Its formation is described as a result of fluid accumulation between the enamel reduced epithelium and the developing tooth crown [7].

Clinically, patients with DC are usually asymptomatic unless the cyst becomes secondarily infected [2,7]. Thus, most of the DCs are discovered fortuitously when radiographs are taken to investigate a tooth eruption failure [7].

Radiographically, most DCs present as a well-defined unilocular radiolucent lesion arising at the cementoenamel junction and surrounding the crown of an impacted tooth [2,4,6].

Many cysts and tumors with radiological appearances related to an embedded tooth may constitute a differential diagnosis challenge for DC. Dental follicle remains the most prominent condition; it can be ruled out as, contrary to DC’s size, it does not exceed 3-4 mm [8]. Odontogenic keratocyst and unicystic ameloblastoma can also be considered; however, a difference exists between these two lesions and DC considering the attachment point to the embedded tooth [9].

Treatment modalities of DC are enucleation and decompression/marsupialization; however, despite the favorable prognosis of DC whatever the surgical technique is, some important factors must be considered for the treatment plan, such as DC size and proximity to anatomic structures, the patient’s age, and the possibility of saving the involved tooth [2,6,10-12]. Therefore, marsupialization in pediatric dentistry was preferred based on the higher tooth eruption potential in children with teeth open apices [12-13].

This report describes a case of a DC associated with a second right mandibular premolar of a 10-year-old girl treated by marsupialization and followed up for five years.

## Case presentation

**Figure 1:**
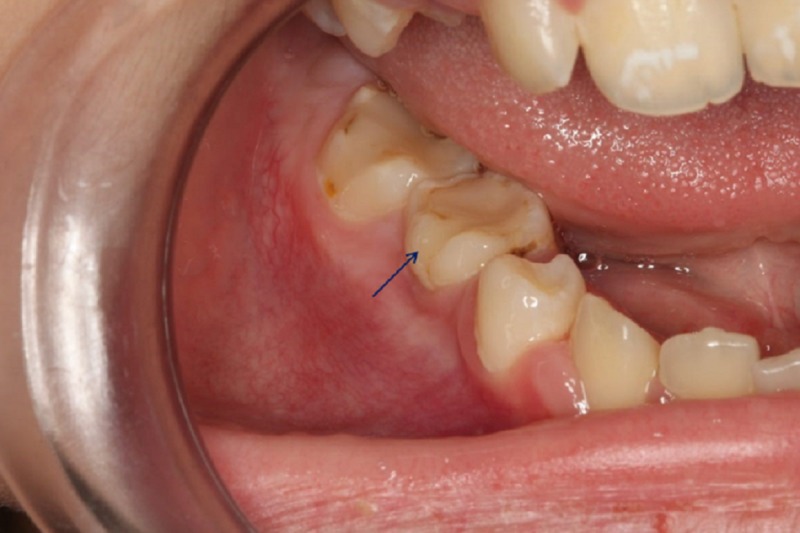
Intraoral photograph Intraoral photograph showing the retention of the second primary molar (blue arrow) with normal overlying mucosa of the vestibule of the right mandibular region

A 10-year-old girl presented to our specialized dental office, along with her parents, complaining from pain when chewing on the right side. Medical and physical examinations revealed a healthy girl with no extra-oral findings. Intra-orally, the right second mandibular premolar was absent, with retention of the right second primary molar. On palpation, moderate pain was felt at the vestibule of the region. The overlying mucosa was normal in color and texture (Figure 1).

**Figure 2:**
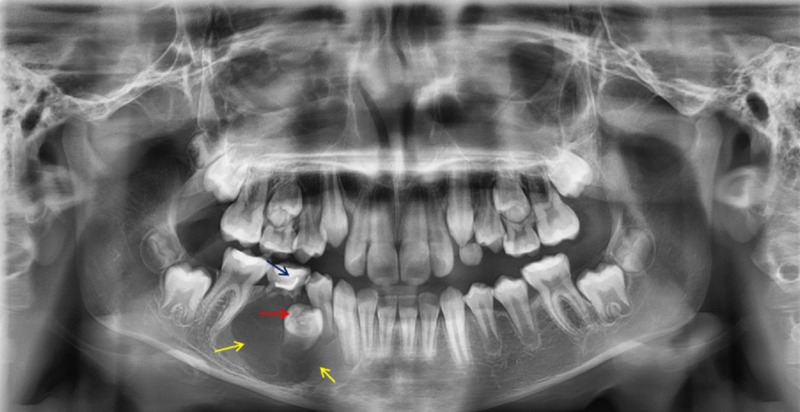
Panoramic radiograph A panoramic radiograph showing a well-defined unilocular radiolucent lesion (yellow arrows) in the right side of the body of the mandible associated with the impacted second premolar (red arrow) and retention of the second primary molar (blue arrow)

No regional lymphadenopathy was noticed. The panoramic radiograph showed a well-defined unilocular radiolucent lesion in the right side of the body of the mandible associated with the impacted second premolar (Figure 2).

Considering the patient’s age, the cyst size and the developmental stage of the involved tooth treatment by marsupialization was effectuated after extraction of the second primary molar.

**Figure 3:**
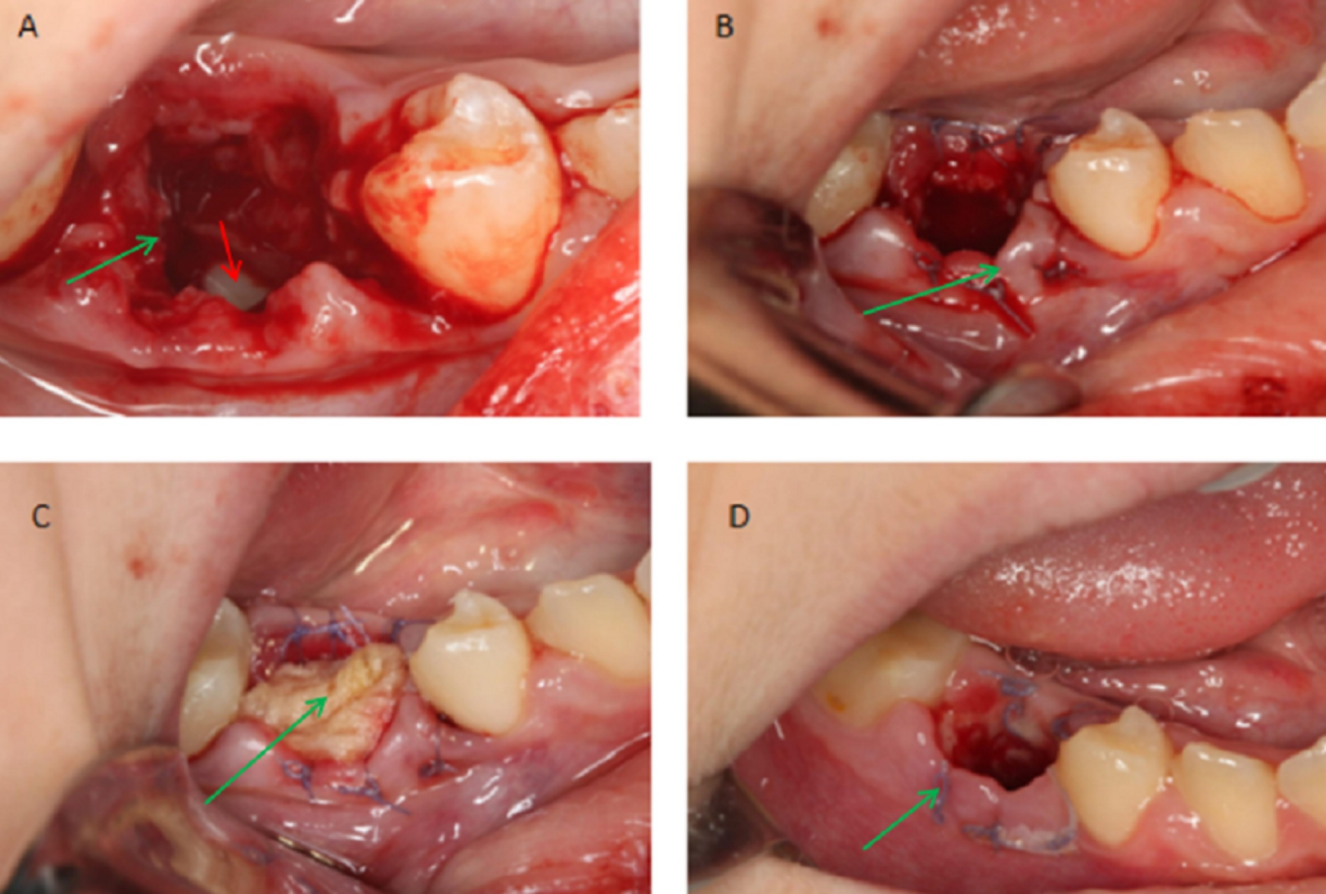
Intraoral photographs Intraoral photographs showing the different steps of the marsupialization (green arrows) after extraction of the second primary molar; to note the position of the involved tooth 45 deep inside the cavity (red arrow)

The process consisted in suturing the edges of the socket induced by the extracted tooth in order to create a communication with the oral cavity allowing a freely continuous draining; sterile gauze was inserted inside the site and replaced weekly (Figure 3).

Histopathologically, the excisional specimen was compatible with a dentigerous cyst.

**Figure 4:**
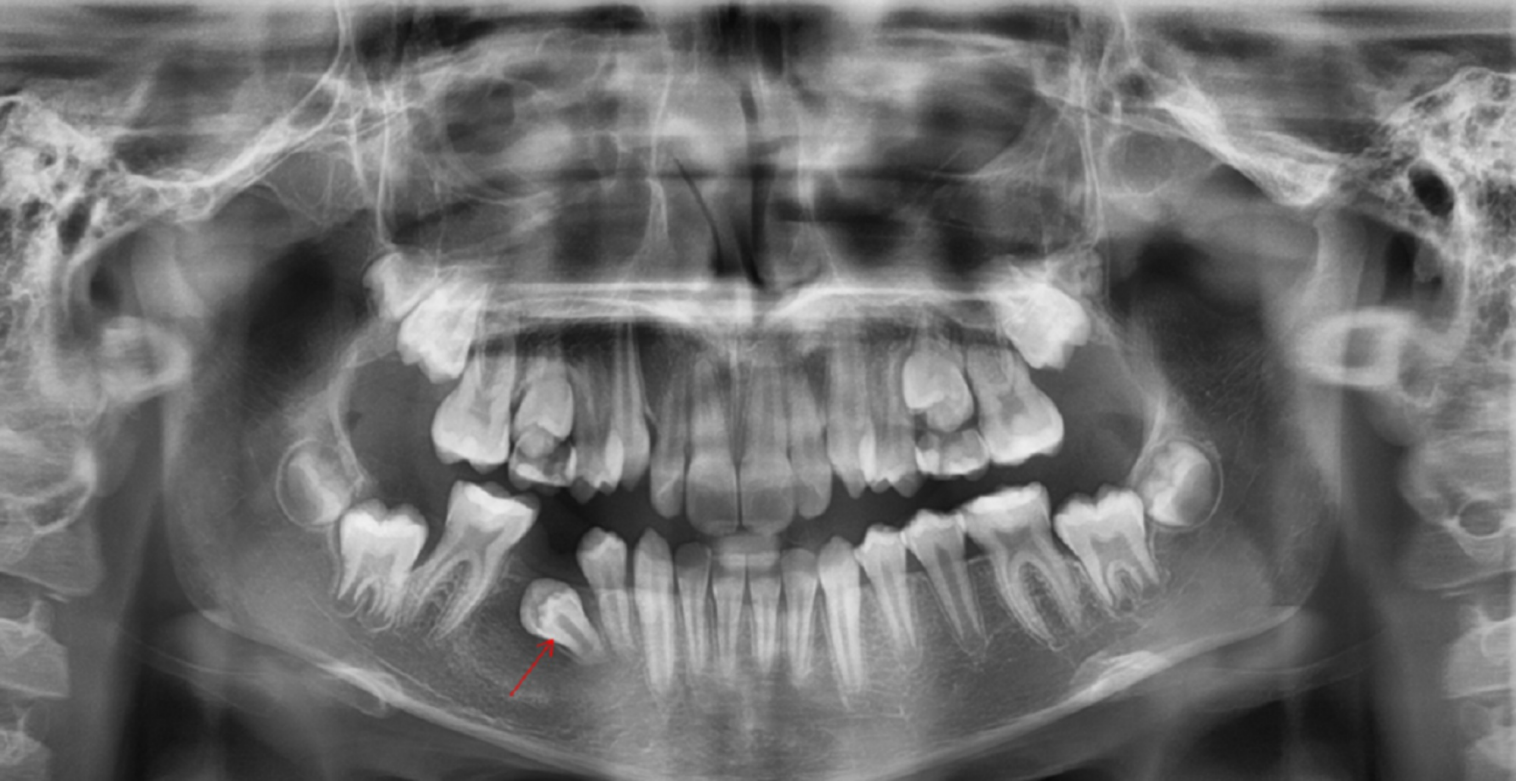
Panoramic radiograph A panoramic radiograph taken after 9 months from the surgery showing regression of the lesion and the new position of the involved tooth 45 (red arrow)

**Figure 5:**
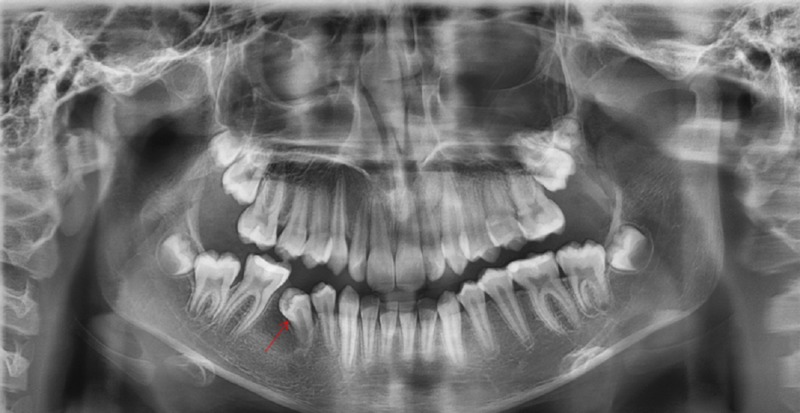
Panoramic radiograph A panoramic radiograph taken after 1 year 8 months from the surgery showing regression of the lesion and the new position of the involved tooth 45 (red arrow).

**Figure 6:**
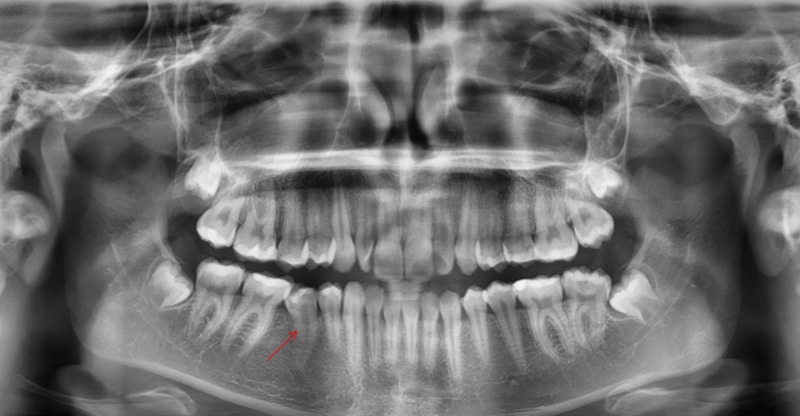
Panoramic radiograph A panoramic radiograph taken after 5 years from the surgery showing complete regression of the lesion and eruption of the involved tooth 45 (red arrow)

The patient showed complete regression of the lesion and eruption of the involved tooth 45; she remained recurrence-free clinically and radiologically after a follow up for a period of 5 years (Figures 4-6).

## Discussion

Although being the most frequent developmental odontogenic cyst to develop in the 2nd (23% of all DCs) and 3rd (20% of all DCs) decades of life, DC can also be found in the 4th, 5th, and 6th decades (17%, 17%, and 12 %, respectively) and less commonly in the 1st and the 7th decades and above [2,4,14-16].

Many authors reported that the DC incidence rate is 14% to 34% of all jaw cysts with 45.7% of them involving the mandibular 3rd molar. Male patients are more affected compared to females; the reason of this sex predilection remains unclear [4,16-18].

The treatment of choice of DC is the enucleation and the removal of the affected tooth; in the case of large cysts, an initial phase of marsupialization followed by the total enucleation is recommended. If DC is connected with a canine or a premolar with a positive eruptive position, marsupialization is preferred [2-3,10-11,12,14].

The prognosis of DC, after successful surgery, is usually excellent with low rate of recurrence.

On the other hand, because of the cases reported about the metaplastic or dysplastic changes of DC and its progression to more severe lesions such as ameloblastoma, mucoepidermoid carcinoma, and squamous cell carcinoma, long-term postoperative surveillance is essential [19-20].

In our case, we have presented a DC related to a second right mandibular premolar of a 10-year-old girl. A conservative treatment by marsupialization has been adopted leading to complete disappearance of the lesion and the eruption of the affected tooth with no recurrence after a five-year follow-up.

## Conclusions

In front of unerupted teeth, thorough clinical and radiological assessments are obligatory in order to investigate the presence of DC. Despite the benignity of these lesions an appropriate treatment must be chosen with a long follow-up period to avoid more complicated situations. Marsupialization is a very effective method in treating DC in children where there is always a chance to save the affected teeth and restores the occlusal functions.
